# Robotic HIPEC with use of a vaginal GelPoint®

**DOI:** 10.1016/j.gore.2024.101400

**Published:** 2024-05-11

**Authors:** Daisy Cruz, Nicole Lugo Santiago, Ernest Han, Jeff Lin

**Affiliations:** a Harbor UCLA Medical Center, United States; b City of Hope Comprehensive Cancer Center, United States; c Weill Cornell Medicine, United States

**Keywords:** HIPEC, GelPoint, Fallopian tube carcinoma

## Abstract

Patients with advanced stage ovarian cancers commonly undergo hyperthermic intraperitoneal chemotherapy (HIPEC) following interval debulking via exploratory laparotomy. This video demonstrates the feasibility of HIPEC delivery via a minimally invasive approach with the use of a vaginal GelPoint® port.

This video demonstrates a 56-year-old patient with Stage 3 bilateral fallopian tube cancer who underwent 3 cycles of neoadjuvant chemotherapy with cisplatin and paclitaxel. Prior to administration of HIPEC the patient underwent an uncomplicated robotic assisted radical hysterectomy, bilateral salpingo-oopherectomy and infracolic omentectomy. Additionally, the falciform ligament was transected.

The vaginal cuff was then used for placement of the GelPoint® port. The inflow and outflow cannulas were placed at the level of the liver and pelvis robotically. To minimize risk of inadvertent spillage, robotic obturators were replaced. Prior to administration of HIPEC, 4 L of warm saline was administered. An additional safety check was performed with no areas of leak. Cisplatin was administered for 90 min followed by sodium thiosulfate and 3 L of normal saline. Confirmation of no residual fluid was noted laparoscopically.

The patient was discharged 2 days postoperatively without postoperative complications.

In this video we demonstrated the innovative technique of performing HIPEC via a minimally invasive approach, that typically requires an open procedure. With the use of a vaginal Gelpoint® we were able to safely administer intraperitoneal chemotherapy without risk to our patient. We were also able to minimize their length of hospital stay and expedite postoperative recovery. Further implementation of this technique may improve hospital resource allocation.

## Highlights

• This video demonstrates gelPoint® port and HIPEC cannula placement.
• The use of a vaginal gelPoint® port for administration of HIPEC expedites patient recovery.
• Vaginal gelPoint® port allows for alternative route of HIPEC administration than traditional exploratory laparotomy.

